# Perceived Difficulty Accessing Primary Health Care and Its Associations With Sociodemographic Factors and Exposure to Violence in a Violent Urban Community in Rio de Janeiro, Brazil: Cross-Sectional Study

**DOI:** 10.2196/94922

**Published:** 2026-09-28

**Authors:** Ana Paula da Cunha, Jurema Corrêa da Mota, Francisco Inacio Bastos

**Affiliations:** 1 Institute of Scientific and Technological Communication and Information in Health, Oswaldo Cruz Foundation, Avenida Brasil, 4365, Rio de Janeiro, Brazil, 55 21992970819; 2 Joaquim Venâncio Polytechnic School of Health, Oswaldo Cruz Foundation, Rio de Janeiro, Brazil; 3 Viral Hepatitis Laboratory, Oswaldo Cruz Institute, Oswaldo Cruz Foundation, Rio de Janeiro, Brazil

**Keywords:** urban violence, primary health care access, sociodemographic characteristics, health care barriers, interpersonal violence

## Abstract

**Background:**

Urban communities affected by chronic violence face persistent challenges in accessing primary health care (PHC). Although violence is widely recognized as a determinant of health inequities, there exists limited evidence on how direct exposure to violent events is related to individuals’ perceptions of difficulty in accessing PHC services in highly vulnerable urban environments.

**Objective:**

The study aims to examine how sociodemographic characteristics and exposure to violent events are associated with perceived difficulty in accessing PHC in standardized hypothetical mental health scenarios among residents of a community affected by chronic urban violence in Rio de Janeiro.

**Methods:**

A cross-sectional survey was conducted between October 8 and November 1, 2024, with 1006 adult residents who completed 4 PHC vignettes and reported past-year exposure to violent events. Participants completed a structured questionnaire that included sociodemographic information, past-year exposure to interpersonal violence, and evaluations of 4 hypothetical vignettes describing different clinical or social situations. Vignette responses were recorded using an ordinal Likert-type scale ranging from “very easy” to “extremely difficult.” Descriptive analyses were performed for all variables, and associations between sociodemographic characteristics and violence indicators were examined using Pearson chi-square tests, with effect sizes estimated using the *φ* coefficient or Cramér *V*, as appropriate, and their respective 95% CIs. Differences in vignette ratings across sociodemographic groups were assessed using Kruskal-Wallis tests. To explore the underlying relationships among violence indicators, vignette responses, and sociodemographic variables, exploratory factor analysis and multiple correspondence analysis were conducted.

**Results:**

Violence exposure was frequent. Threats of harm were reported by 27.7% (157/567) of Black participants and 30.5% (72/236) of non-Black participants. Slapping was reported by 20.8% (118/567) of Black individuals and 28% (66/236) of non-Black individuals, while being pushed or restrained affected 21.2% (50/236) and 26.7% (63/236), respectively. Significant sex differences emerged for being punched or kicked (female 17.2% [75/435] vs male 23.9% [88/368]; *P*=.02). Kruskal-Wallis tests indicated few differences in vignette-based perceptions across groups, except for race in the alcohol-dependence vignette (*χ*²_1_=7.384, *P*=.006). Factor analysis identified 2 dimensions: violence variables loaded strongly on one factor (0.83‐0.96) and vignette responses on another (0.66‐0.83). Multiple correspondence analysis results showed that forms of violence contributed mainly to Dimension 1 (9%‐15%) and vignette categories to Dimension 2 (7%‐12%).

**Conclusions:**

Exposure to violence was widespread, with differences in some forms of violence observed between men and women. Violence-related variables and vignette-based perceptions of difficulty accessing PHC were predominantly represented by distinct dimensions in the exploratory multivariate analyses. Strengthening PHC in violence-affected urban settings requires addressing structural barriers that shape residents’ access to health care.

## Introduction

Urban violence constitutes a major public health challenge and is closely linked to structural inequalities, social exclusion, and territorial segregation in large urban centers. Foundational analyses emphasize that violence functions both as a determinant and an outcome of broader social processes, shaping patterns of illness, emotional distress, and access to essential services [1-7]. These processes disproportionately affect populations living in deprived territories, where state presence is limited and residents routinely navigate environments marked by chronic insecurity. More recent evidence from Rio de Janeiro reinforces the complex relationship between armed violence and adverse living conditions, showing that violence is embedded in broader territorial and social dynamics rather than reflecting a simple linear association with socioeconomic deprivation [8].

These dynamics have historically been particularly evident in favelas and other socioeconomically vulnerable urban territories. Long-term research on the city’s favelas has documented profound transformations in living conditions alongside the persistence of social marginalization, inequality, and violence, demonstrating how improvements in some dimensions of urban life may coexist with enduring processes of social exclusion [9].

In Rio de Janeiro, neighborhoods such as the Manguinhos hub of favelas exemplify how structural inequities intersect with urban violence. Historical accounts and community-based documentation describe Manguinhos as a territory characterized by fragmented urban development, precarious infrastructure, and recurrent episodes of armed conflict, all of which affect the daily lives of residents [10]. The historical formation of Manguinhos has involved heterogeneous processes of occupation, housing interventions, urbanization policies, and disputes over urban space, reflecting broader processes that have shaped the development of favelas in Rio de Janeiro [11]. In this territory, residents experience restricted mobility, environmental hazards, and constant exposure to situations of risk, illustrating how violence shapes daily trajectories, emotional well-being, and opportunities for social participation [12].

In Brazil, PHC is organized within the Unified Health System (Sistema Único de Saúde), primarily through the Family Health Strategy (FHS), a community- and territory-based model in which multidisciplinary teams are responsible for defined populations. The expansion of the FHS has substantially increased access to PHC; however, important barriers persist, including insufficient funding and service capacity, large populations assigned to individual teams, shortages and unequal distribution of health professionals, organizational constraints, and difficulties in ensuring timely and continuous care [13]. These challenges may be particularly relevant in socioeconomically vulnerable territories, where structural and territorial conditions can interact with the organization of health services and further constrain access to care.

The consequences of armed violence in vulnerable territories may also extend to the organization and provision of health care. Health services operating in violence-affected communities may experience disruptions in their activities, while health professionals and residents may face constraints that interfere with the provision and use of care. Recent evidence from vulnerable communities in Rio de Janeiro has shown that armed violence may interact with the functioning of primary health care (PHC) services. In particular, the presence or absence of physicians in PHC teams partially mediated associations between armed violence and selected self-reported health conditions, highlighting the interconnected relationships among territorial violence, health outcomes, and health service organization [14].

International research reinforces the evidence that living in contexts of concentrated poverty and community violence is associated with multiple health consequences, including chronic stress, reduced social cohesion, and behavioral adaptations to perceived danger [15-17]. Studies in diverse global settings demonstrate that residents of high-violence neighborhoods tend to alter their routines, avoid moving through certain areas, and develop heightened perceptions of threat, which may influence their engagement with public services and their interpretations of hypothetical or real situations involving risk or conflict [17]. In the context of PHC, these territorial and social constraints are particularly relevant because access depends not only on the availability of services but also on individuals’ ability to reach, engage with, and use them within the conditions of their everyday lives [18].

Understanding how individuals perceive situations of violence in such contexts is essential for public health. Perception-based data capture dimensions of insecurity, emotional burden, and daily constraints that are not fully captured by official indicators or epidemiological records. Examining how sociodemographic characteristics intersect with reported experiences of violence helps illuminate patterns of vulnerability within territories marked by persistent inequality. Understanding perceptions of access to health care is also relevant in these settings, as perceived barriers may capture dimensions of accessibility that are not fully reflected by indicators of service availability or usage [8,19]. Examining exposure to violence alongside sociodemographic characteristics may therefore help clarify whether direct experiences of violent events are associated with how residents perceive potential difficulties in obtaining PHC.

Despite growing recognition of violence as an important determinant of health and emerging evidence concerning the effects of armed violence on health services in vulnerable urban territories [14], there remains limited evidence on whether individual exposure to interpersonal violence is associated with perceived difficulty in accessing PHC. This distinction is important because the effects of violence on territories and on the organization and functioning of health services do not necessarily imply that individuals who have personally experienced violence perceive greater difficulty in obtaining care. Examining this relationship may help distinguish individual experiences of violence from broader structural, organizational, and territorial barriers to health care access.

The objective of this study is to examine how sociodemographic characteristics and exposure to violent events are associated with perceived difficulty in accessing PHC in standardized hypothetical mental health scenarios, among residents of a community affected by chronic urban violence in Rio de Janeiro.

## Methods

### Study Design and Participants

This is a cross-sectional analytical study including 1006 adults residing in Manguinhos, a community affected by armed violence in Rio de Janeiro, Brazil. The reporting of this study followed the STROBE (Strengthening the Reporting of Observational Studies in Epidemiology) guidelines (Checklist 1). Participants were recruited between October 8 and November 1, 2024, using a quota sampling strategy based on sex and age group to approximate the demographic distribution of the local adult population. A formal a priori power analysis was not performed because the study used a nonprobability quota sampling design nested within a broader population survey. A target of 1000 interviews was established to allow proportional allocation of participants according to sex and age group based on secondary population data provided by the Pereira Passos Institute. The final analytical sample comprised 1006 participants. Recruitment was conducted in public spaces throughout the community by trained interviewers. Individuals were eligible if they were 18 years or older, resided in the study area, and agreed to participate by providing written informed consent. All participants completed a structured questionnaire covering sociodemographic characteristics, exposure to interpersonal violence, and evaluations of 4 vignettes addressing clinical and social conditions.

Quota sampling has limitations compared with probability sampling, particularly regarding representativeness; however, it was chosen to facilitate the recruitment of participants living in an impoverished community with a large proportion of residents in unstable housing (eg, shacks and provisional shelters built with fragile materials) and a highly mobile population. Potential participants were screened for eligibility before the questionnaire was administered. Individuals who did not meet the eligibility criteria were not enrolled, and the electronic questionnaire was programmed to terminate automatically for ineligible respondents. Consequently, all completed questionnaires included in the analyses were obtained from eligible participants.

### Study Variables

Sociodemographic characteristics included sex, age group, and race/color. Exposure to interpersonal violence was assessed with dichotomous questions that asked whether participants had experienced threats, various forms of physical aggression, or attacks involving objects such as knives or firearms in the past year.

Race/color was self-reported according to the Brazilian census classification. For analytical purposes, participants who self-identified as Black or Brown were categorized as Black, whereas those who self-identified as White, Asian, or Indigenous were categorized as non-Black.

Participants also evaluated 4 standardized narrative vignettes describing a hypothetical case of depression and anxiety, a hypothetical case of alcohol dependence, a person with a disadvantaged background but without mental health problems, and a neutral (control) vignette. The complete wording of the vignettes is provided in Multimedia Appendix 1. After reading each vignette, participants answered the following question: “If you had a relative or acquaintance living in the situation described above, how would you rate the level of difficulty they would have in receiving care at the PHC unit serving your neighborhood?” Responses were recorded using a 5-point Likert scale ranging from “very easy” to “extremely difficult,” with an additional “don’t know/no answer” option. The vignette responses were analyzed descriptively and subsequently incorporated into exploratory factor analysis and multiple correspondence analysis (MCA) to investigate the underlying structure of perceived difficulty in accessing PHC.

### Analytical Procedures

Descriptive analyses included absolute and relative frequencies for all variables. Associations between sociodemographic characteristics and violence indicators were examined using Pearson chi-square tests, and the effect size was calculated using the *φ* coefficient or Cramér *V*, with their respective 95% CIs [20].

Differences in vignette ratings according to sex, age, and race/color were analyzed using Kruskal-Wallis tests, given the ordinal distribution of the responses. Although the Kruskal-Wallis test is rank-based and distinct from the chi-square test of independence, its test statistic (*H*) asymptotically follows a chi-square distribution. Therefore, the results are reported using the chi-square statistic (*χ*²) and corresponding *P* values.

### Correlation Matrix and Factor Analysis

A mixed correlation matrix was computed to explore the relationships among vignette responses, violence indicators, and sociodemographic variables. Polychoric correlations were used for ordinal items, tetrachoric correlations for dichotomous variables, and Pearson correlations for numerically recoded sociodemographic variables. This matrix was used as input for the exploratory factor analysis, conducted using principal axis factoring with oblimin rotation [21]. The number of factors was determined based on eigenvalues and parallel analysis. Factor loadings, communalities, and explained variance were examined to support interpretation [22].

### MCA

Given the predominantly categorical nature of the dataset, MCA was subsequently carried out to identify multivariate patterns of association between categories of vignette responses, interpersonal violence indicators, and sociodemographic variables. The analysis produced eigenvalues, explained inertia, category coordinates, squared cosine (cos²) values, and contribution percentages for the main dimensions, which guided the interpretation of the underlying structure.

All analyses were performed in R software (version 4.4.2; R Foundation for Statistical Computing), using the packages *tidyverse* [23], *psych* [24]*, FactoMineR* [25]*, factoextra* [26], and *MASS* [27].

### Ethical Considerations

The study was approved by the Research Ethics Committee of the Oswaldo Cruz Foundation (FIOCRUZ), under CAAE (Certificado de Apresentação para Apreciação Ética [Certificate of Presentation for Ethical Consideration]) number 78822424.9.0000.5241 (approval number 6.873.690). All participants were informed about the study objectives, the voluntary nature of participation, and guarantees of confidentiality. They signed the informed consent form, in accordance with resolution number 466/2012 of the Brazilian National Health Committee on Ethics.

All data were deidentified prior to analysis, and no personally identifiable information was included in the analytical dataset. Participant confidentiality was maintained throughout the study.

Participants did not receive financial compensation for their participation.

## Results

Table 1 presents the distribution of sociodemographic characteristics according to different situations of violence reported in the previous 12 months. For the question “Has someone threatened to harm or injure someone important to you?,” the proportions were similar across race categories: among non-Black participants, 69.5% (164/236) responded “no” and 30.5% (72/236) “yes,” while among Black participants, 72.3% (410/567) responded “no” and 27.7% (157/567) “yes” (*P*=.47, *φ*=0.028, 95% CI 0.000‐0.097). Regarding sex, 72.4% (315/435) of women and 70.4% (259/368) of men reported no such threat (*P*=.58, *φ*=0.022, 95% CI 0.000‐0.103). Across age groups, the distribution also showed similar percentages, with “no” responses ranging from 69.7% (140/201) to 72.7% (208/286) (*P*=.76, Cramer *V*=0.044, 95% CI 0.000‐0.103).

**Table 1.** Distribution of absolute and relative frequencies of sociodemographic variables according to situations of violence, 2024^a^.

Variable and category	Participants, n (%)		*P* value^b^	*ϕ*/Cramer *V* (95% CI)
	No	Yes		
In the past 12 months, has someone threatened to harm or injure someone important to you?				
Race			.47	0.028 (0.000-0.097)
Non-Black	164 (69.5)	72 (30.5)		
Black	410 (72.3)	157 (27.7)		
Sex			.58	0.022 (0.000-0.103)
Female	315 (72.4)	120 (27.6)		
Male	259 (70.4)	109 (29.6)		
Age group (y)			.76	0.044 (0.000-0.103)
18-29	140 (69.7)	61 (30.3)		
30-44	226 (71.5)	90 (28.5)		
45 or older	208 (72.7)	78 (27.3)		
In the past 12 months, has someone slapped you?				
Race			.04	0.078 (0.008-0.146)
Non-Black	170 (72)	66 (28)		
Black	449 (79.2)	118 (20.8)		
Sex			.06	0.069 (0.000-0.138)
Female	347 (79.8)	88 (20.2)		
Male	272 (73.9)	96 (26.1)		
Age group (y)			.02	0.097 (0.008-0.161)
18-29	147 (73.1)	54 (26.9)		
30-44	236 (74.7)	80 (25.3)		
45 or older	236 (82.5)	50 (17.5)		
In the past 12 months, has someone pushed you, grabbed you forcefully, or thrown an object at you with the intention of causing harm?				
Race			.11	0.060 (0.000-0.129)
Non-Black	173 (73.3)	63 (26.7)		
Black	447 (78.8)	120 (21.2)		
Sex			.02	0.084 (0.015-0.153)
Female	350 (80.5)	85 (19.5)		
Male	270 (73.4)	98 (26.6)		
Age group (y)			.36	0.051 (0.000-0.111)
18-29	152 (75.6)	49 (24.4)		
30-44	239 (75.6)	77 (24.4)		
45 or older	229 (80.1)	57 (19.9)		
In the past 12 months, has someone punched, kicked, or pulled you by the hair?				
Race			.14	0.055 (0.000-0.124)
Non-Black	180 (76.3)	56 (23.7)		
Black	460 (81.1)	107 (18.9)		
Sex			.02	0.083 (0.014-0.151)
Female	360 (82.8)	75 (17.2)		
Male	280 (76.1)	88 (23.9)		
Age group (y)			.09	0.078 (0.000-0.141)
18-29	155 (77.1)	46 (22.9)		
30-44	245 (77.5)	71 (22.5)		
45 or older	240 (83.9)	46 (16.1)		
In the past 12 months, has someone attempted or actually strangled, suffocated, or intentionally burned you?				
Race			.12	0.059 (0.000-0.128)
Non-Black	204 (86.4)	32 (13.6)		
Black	513 (90.5)	54 (9.5)		
Sex			.48	0.029 (0.000-0.098)
Female	392 (90.1)	43 (9.9)		
Male	325 (88.3)	43 (11.7)		
Age group (y)			.12	0.073 (0.000-0.136)
18-29	177 (88.1)	24 (11.9)		
30-44	276 (87.3)	40 (12.7)		
45 or older	264 (92.3)	22 (7.7)		
In the past 12 months, has someone threatened or injured you with a knife, firearm, or any other weapon or object?				
Race			.22	0.047 (0.000-0.116)
Non-Black	206 (87.3)	30 (12.7)		
Black	513 (90.5)	54 (9.5)		
Sex			.35	0.037 (0.000-0.106)
Female	394 (90.6)	41 (9.4)		
Male	325 (88.3)	43 (11.7)		
Age group (y)			.10	0.076 (0.000-0.139)
18-29	176 (87.6)	25 (12.4)		
30-44	278 (88)	38 (12)		
45 or older	265 (92.7)	21 (7.3)		

a The *φ* coefficient was calculated for the race and sex variables, whereas Cramér *V* was calculated for age group.

b Chi-square test.

A high proportion of both Black and non-Black participants reported not having been slapped: 72.0% (170/236) of non-Black individuals and 79.2% (449/567) of Black individuals answered “no” (*P*=.04, φ=0.078, 95% CI 0.008‐0.146). Among women, 79.8% (347/435) reported not having been slapped, compared to 73.9% (272/368) of men (*P*=.06, *φ*=0.069, 95% CI 0.000‐0.138). Across age groups, the proportion of “no” responses varied from 73.1% (147/201) to 82.5% (236/286; *P*=.02, Cramer *V*=0.097, 95% CI 0.008‐0.161; Table 1).

For being pushed or grabbed forcefully, or having an object thrown with the intention of causing harm, 73.3% (173/236) of non-Black individuals and 78.8% (447/567) of Black participants reported “no” (*P*=.11, *φ*=0.060, 95% CI 0.000‐0.129). Women reported not experiencing this event in 80.5% (350/435) of cases compared with 73.4% (270/368) of men (*P*=.02, *φ*=0.084, 95% CI 0.015‐0.153). Across age groups, “no” responses ranged from 75.6% (152/201) to 80.1% (229/286; *P*=.36, Cramer*V*=0.051, 95% CI 0.000‐0.111; Table 1).

Physical aggression involving punching, kicking, or pulling hair was not reported by 76.3% (180/236) of non-Black participants and 81.1% (460/567) of Black participants (*P*=.14, *φ*=0.055, 95% CI 0.000‐0.124). A difference between sexes was observed in the proportions of “yes” responses: 17.2% (75/435) among women and 23.9% (88/368) among men (*P*=.02, *φ*=0.083, 95% CI 0.014‐0.151). Across age groups, “yes” responses ranged from 16.1% (46/286) to 22.9% (46/201) across groups (*P*=.09, Cramer *V*=0.078, 95% CI 0.000‐0.141; Table 1).

Most participants reported not experiencing attempted or actual strangulation, suffocation, or burning in the previous 12 months. This proportion was slightly higher among Black participants (513/567, 90.5%) than among non-Black participants (204/236, 86.4%), although the difference was not statistically significant (*P*=.12, *φ*=0.059, 95% CI 0.000‐0.128). A similar pattern was observed by sex: 90.1% (392/435) of women and 88.3% (325/368) of men reported not experiencing these acts (*P*=.48, *φ*=0.029, 95% CI 0.000‐0.098). Across age groups, the proportion reporting no such violence ranged from 87.3% (276/316) to 92.3% (264/286; *P*=.12, Cramer *V*=0.073, 95% CI 0.000‐0.136; Table 1).

For threats or injuries involving a knife, firearm, or another weapon, 87.3% (206/236) of non-Black participants and 90.5% (513/567) of Black participants responded “no” (*P*=.22, *φ*=0.047, 95% CI 0.000‐0.116). Women reported not experiencing this type of violence in 90.6% (394/435) of cases, compared with 88.3% (325/368; *P*=.35, *φ*=0.037, 95% CI 0.000‐0.106). Across age groups, “no” responses ranged from 87.6% (176/201) to 92.7% (265/286; *P*=.10, Cramer *V*=0.076, 95% CI 0.000‐0.139; Table 1).

Sex comparisons revealed similar vignette response scores for the depression vignette (*χ*²_1_=0.51, *P*=.48), the alcohol-dependence vignette (*χ*²_1_=0.04, *P*=.84), the vignette without mental health problems (*χ*²_1_=0.15, *P*=.70), and the control vignette (*χ*²_1_=0.01, *P*=.91; Table 2).

**Table 2.** Comparison of vignette response scores by sex, race/color, and age group.

Grouping variable and vignette	Chi-square (*df*) statistic	*P* value
Sex		
Depression vignette	0.508 (1)	.48
Alcohol-dependence vignette	0.041 (1)	.84
No mental health problems vignette	0.146 (1)	.70
Control vignette	0.013 (1)	.91
Race/color		
Depression vignette	0.009 (1)	.92
Alcohol-dependence vignette	7.384 (1)	.006
No mental health problems vignette	3.140 (1)	.08
Control vignette	0.429 (1)	.51
Age group		
Depression vignette	1.565 (2)	.46
Alcohol-dependence vignette	1.561 (2)	.46
No mental health problems vignette	4.340 (2)	.11
Control vignette	0.125 (2)	.94

Race and color comparisons revealed similar vignette response scores for the depression vignette (*χ*²_1_=0.01, *P*=.92), the vignette without mental health problems (*χ*²_1_=3.14, *P*=.08), and the control vignette (*χ*²_1_=0.43, *P*=.51). A statistically significant difference was observed only for the alcohol-dependence vignette (*χ*²_1_=7.38, *P*=.006; Table 2).

Age-group comparisons revealed similar vignette response scores for the depression vignette (*χ*²_2_=1.57, *P*=.46), the alcohol-dependence vignette (*χ*²_2_=1.56, *P*=.46), the vignette without mental health problems (*χ*²_2_=4.34, *P*=.11), and the control vignette (*χ*²_2_=0.13, *P*=.94; Table 2).

Table 3 presents the factor loadings for the 2-factor solution derived from principal axis factoring with oblimin rotation. The 4 vignette items loaded more strongly on Factor 2, with values of 0.73 for the depression vignette, 0.83 for the alcohol-dependence vignette, 0.80 for the vignette without mental health problems, and 0.66 for the control vignette.

**Table 3.** Factor loadings^a^ for a 2-factor solution.

Variable	Factor 1 (PA1^b^)	Factor 2 (PA2)	*h*²
Depression vignette	0.05	0.73	0.54
Alcohol-dependence vignette	0.00	0.83	0.70
No mental health problems vignette	−0.02	0.80	0.63
Control vignette	−0.04	0.66	0.43
Threatened to hurt or injure someone important	0.83	0.02	0.69
Slapped (past 12 months)	0.94	0.05	0.89
Pushed/held down (past 12 months)	0.95	−0.02	0.90
Punched/kicked (past 12 months)	0.96	0.00	0.92
Strangled/suffocated/burned (past 12 months)	0.92	−0.05	0.85
Threatened/attacked with a weapon	0.84	−0.01	0.71
Sex	0.10	0.11	0.02
Age group	−0.10	−0.12	0.03
Race/color	−0.08	−0.02	0.01

a Factor loadings were obtained using principal axis factoring with oblimin rotation.

b PA: principal axis.

All 6 violence-related items loaded more strongly on Factor 1, with loadings ranging from 0.83 (threatened to harm or injure someone important) to 0.96 (punched or kicked in the past 12 months). The variable “threatened/attacked with a weapon” showed a loading of 0.84 on Factor 1, while “strangled/suffocated/burned” loaded 0.92 on the same factor (Table 3).

Sociodemographic variables showed low loadings on both factors, with values of 0.10 and 0.11 for sex, –0.10 and –0.12 for age group, and –0.08 and –0.02 for race/color. Communality values (*h*²) ranged from 0.01 (race/color) to 0.92 (punched/kicked in the past 12 months; Table 3).

Table 4 displays the squared cosine (cos²) values and the percentage contributions of each category to Dimensions 1 and 2 of the MCA. Across the violence-related variables, high cos² values on Dimension 1 were observed for the “yes” and “no” categories of punched/kicked (0.745), slapped (0.717), pushed/restrained (0.699), strangled/suffocated/burned (0.563), threatened or injured with a weapon (0.520), and threatened to hurt someone important (0.507). These categories also made the largest contributions to Dimension 1, with values ranging from 9.01% to 14.75% for the “yes” categories.

**Table 4.** Squared cosine (cos²) and dimension contributions (%) for each variable category in the multiple correspondence analysis (MCA).

Variable and category	Cos²		Contribution	
	Dimension 1	Dimension 2	Dimension 1 (%)	Dimension 2 (%)
Punched/kicked				
Yes	0.745	0.016	14.75	0.45
No	0.745	0.016	3.76	0.11
Slapped				
Yes	0.717	0.021	13.73	0.59
No	0.717	0.021	4.08	0.18
Pushed/restrained				
Yes	0.699	0.038	13.41	1.07
No	0.699	0.038	3.96	0.32
Strangled/suffocated/burned				
Yes	0.563	0.028	12.48	0.92
No	0.563	0.028	1.50	0.11
Threatened/injured with a weapon				
Yes	0.520	0.026	11.56	0.85
No	0.520	0.026	1.35	0.10
Threatened to hurt someone important				
Yes	0.507	0.023	9.01	0.60
No	0.507	0.023	3.59	0.24
Depression vignette				
Very easy	0.017	0.335	0.39	11.43
Easy	0.014	0.251	0.31	8.18
Moderate	0.009	0.004	0.18	0.12
Very difficult	0.022	0.129	0.35	2.96
Extremely difficult	0.007	0.041	0.13	1.15
Alcohol-dependence vignette				
Very easy	0.018	0.353	0.43	12.27
Easy	0.031	0.289	0.67	9.25
Moderate	0.000	0.003	0.00	0.08
Very difficult	0.030	0.151	0.46	3.33
Extremely difficult	0.000	0.040	0.00	1.18
No mental health problems vignette				
Very easy	0.019	0.343	0.44	11.87
Easy	0.026	0.216	0.57	6.96
Moderate	0.000	0.000	0.01	0.00
Very difficult	0.036	0.149	0.58	3.49
Extremely difficult	0.000	0.050	0.00	1.49
Control vignette				
Very easy	0.023	0.316	0.54	10.76
Easy	0.022	0.135	0.47	4.23
Moderate	0.003	0.001	0.06	0.01
Very difficult	0.020	0.155	0.33	3.64
Extremely difficult	0.001	0.032	0.01	1.00
Sex				
Female	0.011	0.013	0.12	0.21
Male	0.011	0.013	0.14	0.25
Age group (y)				
18‐29	0.004	0.012	0.08	0.33
30‐44	0.004	0.000	0.06	0.01
≥45	0.015	0.007	0.25	0.15
Race/color				
Non-Black	0.011	0.004	0.19	0.10
Black	0.011	0.004	0.08	0.04

For the vignette response categories, the highest cos² values on Dimension 2 were observed for the “very easy” and “easy” categories across all 4 vignettes. In the depression vignette, cos² values were 0.335 and 0.251; in the alcohol-dependence vignette, 0.353 and 0.289; in the vignette without mental health problems, 0.343 and 0.216; and in the control vignette, 0.316 and 0.135. These categories also showed the highest contributions to Dimension 2, ranging from 6.96% to 12.27% (Table 4).

Sociodemographic variables showed low cos² values in both dimensions. For sex, cos² values were 0.011 for Dimension 1 and 0.013 for Dimension 2, with contributions below 0.25%. Age groups showed cos² values ranging from 0.004 to 0.015 for Dimension 1 and from 0.000 to 0.012 for Dimension 2. Race/color categories showed cos² values of 0.011 on Dimension 1 and 0.004 on Dimension 2, with contributions below 0.20% (Table 4).

## Discussion

The findings of this study indicate that exposure to interpersonal violence was frequent in the study population, while perceptions regarding the difficulty of the clinical and social situations described in the vignettes formed a distinct analytical dimension from violence exposure. In addition, perceived difficulty in accessing PHC showed limited variation across sociodemographic groups. This separation was consistently observed across exploratory multivariate analyses, including factor analysis and MCA, which revealed 2 clearly differentiated dimensions: one associated with violence-related experiences and another associated with perceived difficulty accessing PHC as assessed through the vignette scenarios. Differences between experiences and perceptions under stressful conditions have been documented in the psychological and public health literature. As discussed by Krieger [28], such challenging “crossroads” call for further theoretical development and innovative empirical studies.

Discrepancies between actual experiences of violence and individual or community perceptions of violence have been documented across diverse settings, ranging from local conflicts, such as turf wars [29], to large-scale and protracted conflicts [30]. Such differences in perceptions and responses may contribute to variation in psychological responses to adversity, including posttraumatic stress and resilience. However, resilience may be challenged by prolonged or cumulative exposure to adversity, as illustrated by recent analyses of the combined effects of war and environmental stressors [31].

The prevalence levels observed in the sample are consistent with global patterns of physical violence and threats, as documented in international epidemiological studies. Evidence from large multicountry surveys has repeatedly shown that exposure to physical or psychological aggression is widespread across different populations and settings [32-36].

Although most comparisons did not indicate statistically significant sociodemographic differences, the distribution of violence types within groups was broadly aligned with the international literature. Men reported higher frequencies of some forms of aggression, consistent with studies showing that men experience substantial levels of nonpartner physical violence, especially in community or nondomestic contexts [37-40].

At the same time, women reported higher exposure in certain categories in other international surveys, particularly those involving coercive control or severe violence [36,41]. Such patterns reinforce the notion that interpersonal violence is multifaceted and extends beyond intimate-partner contexts.

Limited variation was observed in vignette-response scores by sex, race/color, or age group, with only one statistically significant result (race/color in the alcohol-dependence vignette). International studies using vignette-based approaches have similarly documented variation in how individuals evaluate standardized health-related scenarios across sociodemographic groups [3,42].

Factor analysis results demonstrated a clear 2-factor structure: violence-related items loaded strongly on the first factor, and vignette-evaluation items loaded on the second, with minimal loadings of sociodemographic variables. This separation suggests that, in this sample, previous exposure to interpersonal violence and perceived difficulty accessing PHC through the vignette scenarios represented distinct analytical dimensions [36].

The MCA results were consistent with this distinction, with “yes” categories for violence defining Dimension 1 and vignette categories defining Dimension 2. Similar patterns of violence clustering have been reported in multicountry studies examining violence and psychosocial variables [37,43,44].

Taken together, the results suggest that violence exposure and perceptions of difficulty accessing PHC appear to represent distinct dimensions in this sample. International literature indicates that interpersonal violence is a strong predictor of mental-health outcomes such as depression, anxiety, posttraumatic stress disorder, and suicidality [33,36,38]. These findings highlight the importance of distinguishing previous exposure to interpersonal violence from perceptions of difficulty accessing PHC, as both may be shaped by different individual, organizational, and contextual factors [45-48].

The limited differentiation in vignette responses across sociodemographic groups also suggests that perceived difficulty accessing PHC cannot be attributed to demographic characteristics alone. Broader structural, organizational, and territorial factors should therefore be considered when developing strategies to improve access to PHC in violence-affected communities [36,37,49,50].

Overall, the results indicate that, although exposure to violence was common and patterned in ways broadly consistent with international evidence, violence-related variables and vignette-based perceptions of difficulty accessing PHC were predominantly represented by distinct dimensions in the exploratory multivariate analyses. This distinction underscores the importance of considering perceived access as a multidimensional construct that may be influenced by factors beyond individual experiences of violence.

These findings also have important implications for the organization of PHC in violence-affected territories. The distinction observed between violence-related variables and vignette-based perceptions of difficulty accessing PHC in the exploratory multivariate analyses highlights the need to consider broader structural, organizational, and social factors that may shape perceived access. Consequently, strategies aimed at improving access should extend beyond the identification of violence exposure and include actions to strengthen service availability, continuity of care, welcoming practices, and coordination of care within PHC.

In the Brazilian context, these implications should be considered in relation to the organization of PHC and the structural conditions affecting access to care. Although the expansion of the FHS has contributed substantially to increased PHC coverage and access, persistent organizational and structural barriers may limit the ability of services to provide timely and continuous care, particularly in socially vulnerable territories [13]. In settings affected by chronic armed violence, these barriers may be compounded by disruptions in service functioning, restrictions on the mobility of residents and health professionals, and difficulties in maintaining stable health care teams. Recent evidence from impoverished communities in Rio de Janeiro has shown that armed violence can affect PHC functioning, with the presence or absence of physicians in primary care teams partially mediating the associations between exposure to armed violence and selected health conditions [14]. Thus, perceptions of difficulty in accessing PHC in Manguinhos should be interpreted not solely in relation to individual characteristics, but also within the broader context of territorial violence and the structural and organizational conditions that may shape access to health services.

However, the observed distinction between violence-related variables and vignette-based perceptions of difficulty accessing PHC should be interpreted with caution. It should not be interpreted as evidence that violence has no influence on perceived access to PHC. Rather, it may reflect the multidimensional nature of access, which is influenced by organizational characteristics of health services, previous experiences with care, social support, and contextual factors that were not directly assessed in this study.

This study has some limitations. First, its cross-sectional design precludes causal inference. Second, the outcome was based on responses to standardized hypothetical vignettes, which reflect perceived difficulty in accessing PHC rather than actual health care usage or experienced barriers to access. Although the vignettes were designed to assess perceived difficulty accessing PHC rather than mental health literacy, responses to hypothetical mental health scenarios may also be influenced by participants’ beliefs and understanding of mental health conditions, which have been shown to vary within community populations. Third, although the analyses included key sociodemographic characteristics, other potentially relevant contextual and socioeconomic determinants of access, such as education, income, employment status, previous experiences with health services, and geographic accessibility, were not evaluated. These factors may influence perceived difficulty accessing PHC and should be considered in future studies using multivariable analytical approaches.

Furthermore, the distinction observed between violence-related variables and vignette-based perceptions of difficulty accessing PHC in the exploratory multivariate analyses should be interpreted with caution. This pattern should not be interpreted as evidence of the absence of an association between violence exposure and perceived difficulty accessing PHC. Future studies using complementary measures of access and multivariable analytical approaches may help clarify this relationship.

Understanding the factors that shape perceptions of access to PHC is essential for strengthening health systems in communities affected by chronic urban violence. Although violence-related variables and vignette-based perceptions of difficulty accessing PHC were predominantly represented by distinct dimensions in the exploratory multivariate analyses, the findings suggest that broader structural, organizational, and social determinants deserve greater attention in policies and interventions aimed at improving access. Future studies conducted in different contexts and using complementary measures of access are needed to further clarify these relationships and support the development of more equitable PHC services.

## Supplementary material

10.2196/94922 Multimedia Appendix 1 Full wording of the standardized vignettes used in the study.

10.2196/94922 Checklist 1 STROBE checklist for cross-sectional studies.

## Abbreviations

CAAE: Certificado de Apresentação para Apreciação Ética (Certificate of Presentation for Ethical Consideration)
FHS: Family Health Strategy
MCA: multiple correspondence analysis
PHC: primary health care
STROBE: Strengthening the Reporting of Observational Studies in Epidemiology

## References

[1] Minayo MCS (1994). Social violence from a public health perspective. Cad Saude Publica.

[2] Hollie BD, Coolhart D (2020). “A larger system is placing people in this predicament”: a qualitative exploration of living amongst urban violence and the impact on mental health and relationships in the Black community. Contemp Fam Ther.

[3] Borg BA, Rabinak CA, Marusak HA (2023). Violence exposure and mental health consequences among urban youth. Curr Psychol.

[4] Coid J, Zhang Y, Ullrich S (2021). Interpersonal violence in a deprived Scottish urban area with aggregations of physical health risks and psychiatric morbidity: an ecological study. BMC Public Health.

[5] Green G, Swartz A, Tembo D (2023). A scoping review of how exposure to urban violence impacts youth access to sexual, reproductive and trauma health care in LMICs. Glob Public Health.

[6] Pérez-Sastré MA, García-Peña C, Ramos-Lira L, Ortiz-Hernández L (2024). Beyond direct exposure to violence: effects of living in disordered and violent communities on psychological distress in young Mexican people. Cad Saude Publica.

[7] Vieira-Meyer A, Vieira NF, Machado FA (2025). Urban violence and its effect on primary health care. Eur J Public Health.

[8] da Cunha AP, Corrêa da Mota J, Bastos FI (2026). A city under siege: time-series analysis of shootouts and the association with health and living conditions in Rio de Janeiro, Brazil, 2017 to 2022. J Interpers Violence.

[9] Perlman J (2010). Favela: Four Decades of Living on the Edge in Rio de Janeiro.

[10] Dicionário de Favelas Marielle Franco (2024). Favela de manguinhos [Article in Portuguese]. Wikifavelas.

[11] Fernandes TM, Costa RGR (2013). As comunidades de Manguinhos na história das favelas no Rio de Janeiro. Tempo.

[12] Lyra D (2012). Infância e trânsito em manguinhos [Report in Portuguese]. https://www.cecip.org.br/site/wp-content/uploads/2016/08/Manguinhos-e-seus-Caminhos-relatorioCPFoco.pdf.

[13] Tesser CD, Norman AH, Vidal TB (2018). Acesso ao cuidado na Atenção Primária à Saúde brasileira: situação, problemas e estratégias de superação [Article in Portuguese]. Saúde debate.

[14] Mota JCD, Cunha APD, Bastos FI (2025). Armed violence and shootouts in Rio de Janeiro: relevance to impoverished communities and primary care health services. Cien Saude Colet.

[15] Sampson RJ (2012). Great American City: Chicago and the Enduring Neighborhood Effect.

[16] Aiyer SM, Zimmerman MA, Morrel-Samuels S, Reischl TM (2015). From broken windows to busy streets: a community empowerment perspective. Health Educ Behav.

[17] Krug EG, Dahlberg LL, Mercy JA, Zwi AB, Lozano R (2002). World report on violence and health. https://iris.who.int/server/api/core/bitstreams/a25476ed-8585-47f3-986e-9d0e7f5e9f1b/content.

[18] Levesque JF, Harris MF, Russell G (2013). Patient-centred access to health care: conceptualising access at the interface of health systems and populations. Int J Equity Health.

[19] Topbas M, Dilaver I, Karakullukcu S (2026). What influences the perceived access to healthcare services? An investigation using structural equation modeling. Front Public Health.

[20] Ferguson CJ (2009). An effect size primer: a guide for clinicians and researchers. Prof Psychol Res Pract.

[21] Ekström J (2011). A generalized definition of the polychoric correlation coefficient. https://escholarship.org/content/qt583610fv/qt583610fv.pdf?v=lg.

[22] Jackson JE, Armitage P, Colton T (2005). Encyclopedia of Biostatistics.

[23] Wickham H, Averick M, Bryan J (2019). Welcome to the tidyverse. J Open Source Softw.

[24] Revelle W (2026). Psych: procedures for psychological, psychometric, and personality research. R package version 2.6.1. CRAN (Comprehensive R Archive Network).

[25] Lê S, Josse J, Husson F (2008). FactoMineR: an R package for multivariate analysis. J Stat Soft.

[26] Kassambara A, Mundt F, Erdey L (2026). Factoextra: extract and visualize the results of multivariate data analyses. CRAN (Comprehensive R Archive Network).

[27] Venables WN, Ripley BD (2002). Modern Applied Statistics with S.

[28] Krieger N (2016). Living and dying at the crossroads: racism, embodiment, and why theory is essential for a public health of consequence. Am J Public Health.

[29] Modan GG (2007). Turf Wars: Discourse, Diversity, and the Politics of Place.

[30] Litz BT (2014). Resilience in the aftermath of war trauma: a critical review and commentary. Interface Focus.

[31] Döring S, Hecker T, Hall J (2026). Drought amplifies the psychological burden of war. Nat Sustain.

[32] Garcia-Moreno C, Jansen HA, Ellsberg M, Heise L, Watts CH (2006). Prevalence of intimate partner violence: findings from the WHO multi-country study on women’s health and domestic violence. Lancet.

[33] Ellsberg M, Jansen H, Heise L, Watts CH, Garcia-Moreno C (2008). Intimate partner violence and women's physical and mental health in the WHO multi-country study on women's health and domestic violence: an observational study. Lancet.

[34] Sardinha L, Maheu-Giroux M, Stöckl H, Meyer SR, García-Moreno C (2022). Global, regional, and national prevalence estimates of physical or sexual, or both, intimate partner violence against women in 2018. Lancet.

[35] Clark CJ, Bergenfeld I, Cheong YF (2023). Patterns of women’s exposure to psychological violence: a global examination of low- and middle-income countries. SSM Popul Health.

[36] White SJ, Sin J, Sweeney A (2024). Global prevalence and mental health outcomes of intimate partner violence among women: a systematic review and meta-analysis. Trauma Violence Abuse.

[37] Abramsky T, Watts CH, Garcia-Moreno C (2011). What factors are associated with recent intimate partner violence? Findings from the WHO multi-country study on women’s health and domestic violence. BMC Public Health.

[38] Fanslow JL, Malihi Z, Hashemi L, Gulliver P, McIntosh T (2022). Prevalence of interpersonal violence against women and men in New Zealand: results of a cross-sectional study. Aust N Z J Public Health.

[39] Scott-Storey K, O’Donnell S, Ford-Gilboe M (2023). What about the men? A critical review of men's experiences of intimate partner violence. Trauma Violence Abuse.

[40] Fanslow JL, Gulliver PJ, Mellar BM, Selak V, Mcintosh TKD (2025). Men’s lifetime interpersonal violence exposure and associations with hospitalization at the national level: results from a New Zealand population-based study. Scand J Public Health.

[41] Kifle ME, Aychiluhm SB, Anbesu EW (2024). Global prevalence of intimate partner violence during the COVID-19 pandemic among women: systematic review and meta-analysis. BMC Womens Health.

[42] Ciciurkaite G, Pescosolido BA (2024). Mental health literacy and public stigma: examining the link in 17 countries. Med Res Arch.

[43] Jewkes R, Jordaan E, Myrttinen H, Gibbs A (2020). Masculinities and violence: using latent class analysis to investigate the origins and correlates of differences between men in the cross-sectional UN Multi-country Study on Men and Violence in Asia and the Pacific. J Glob Health.

[44] Pettersson R, Lucas S, Strandh M (2025). Patterns of violence exposure in a life-course perspective and associations to mental and physical health problems and health-related risk behaviors among women and men in Sweden: a latent class analysis. SSM Popul Health.

[45] Ogbe E, Harmon S, Van den Bergh R, Degomme O (2020). A systematic review of intimate partner violence interventions focused on improving social support and/ mental health outcomes of survivors. PLoS One.

[46] Paphitis SA, Bentley A, Asher L, Osrin D, Oram S (2022). Improving the mental health of women intimate partner violence survivors: findings from a realist review of psychosocial interventions. PLoS One.

[47] Mellen EJ, Hatzenbuehler ML (2023). Sexual violence-related stigma, mental health, and treatment-seeking: a multimodal assessment in a population-based study of young adults. J Interpers Violence.

[48] Mellen EJ, Kim DY, Edenbaum ER, Cellini J (2025). The psychosocial consequences of sexual violence stigma: a scoping review. Trauma Violence Abuse.

[49] Bhavsar V, Bhugra D, Persaud A (2020). Public policy, mental health and a public health approach to violence. Int J Soc Psychiatry.

[50] Oram S, Fisher HL, Minnis H (2022). The Lancet Psychiatry Commission on intimate partner violence and mental health: advancing mental health services, research, and policy. Lancet Psychiatry.

