# Supplementary material for: Perceived Difficulty Accessing Primary Health Care and Its Associations With Sociodemographic Factors and Exposure to Violence in a Violent Urban Community in Rio de Janeiro, Brazil: Cross-Sectional Study
Source: JMIR Public Health Surveill. 2026 Sep 28;12:e94922. doi: 10.2196/94922 (PMC13618411; doi:10.2196/94922)
Supplement: Supplementary file 1 [file publichealth-v12-e94922-s001.pdf]

Vignettes

Vignette 1. Typical history of depression and anxiety

José, a 26-year-old man, has mental health problems that are controlled with medication for depression. However, whenever he starts feeling better, he stops taking his medication without medical advice, which leads to inappropriate behaviors such as theft and aggression. He lives alone but maintains close contact with family and friends, who monitor and support him whenever they notice signs of relapse. Despite these difficulties, José has good personal and professional relationships, although interrupting his treatment negatively affects his behavior and social interactions.

Question: If you had a relative or acquaintance living in the situation described above, how would you rate the level of difficulty they would have in receiving care at the primary health care unit serving your neighborhood?

Vignette 2. Typical history of alcohol dependence

José, a 26-year-old man, has struggled with alcohol dependence since graduating from college, using alcohol as a way of coping with difficult situations. Under the influence of alcohol, he becomes involved in theft and disturbs the neighborhood with unpredictable behavior, alternating between being outgoing and withdrawn. He lives alone but maintains close contact with family and friends, who support him whenever they recognize signs of relapse. Although he has a good educational background and stable personal relationships, alcohol dependence negatively affects his behavior and social interactions. His support network actively helps him maintain control over his condition.

Question: *If you had a relative or acquaintance living in the situation described above, how would you rate the level of difficulty they would have in receiving care at the primary health care unit serving your neighborhood?*

Vignette 3. Disadvantaged background without mental health problems

Maria, a 26-year-old woman, has a history of disruptive behavior, including theft and causing disturbances in her neighborhood. She lives alone and has only intermittent contact with her family, maintaining a closer relationship only with her half-sister. Maria's childhood was marked by family conflict, periods spent in orphanages, and exposure to domestic violence, all of which affected her self-esteem and sense of identity. Although she performed well academically, she left college early and now works in retail while maintaining few close relationships. These adverse life experiences have shaped her behavioral difficulties, and she tends to direct her frustrations toward family members, despite the ongoing support of her half-sister, who provides an important source of stability in her life.

Question: *If you had a relative or acquaintance living in the situation described above, how would you rate the level of difficulty they would have in receiving care at the primary health care unit serving your neighborhood?*

Vignette 4. Control vignette (without distinctive characteristics)

Maria, a 26-year-old woman, lives alone in a rented room but maintains regular and close contact with her immediate family. She has a good relationship with her parents and siblings and frequently stays overnight at her brother's home. Apart from the usual disagreements during adolescence, Maria has positive memories of her childhood. She graduated in Computer Science after an excellent academic career and currently works in her field of training. She is sociable and has maintained stable relationships since childhood, which contribute positively to her self-esteem and sense of identity, reinforced by supportive family relationships and positive life experiences.

Question: *If you had a relative or acquaintance living in the situation described above, how would you rate the level of difficulty they would have in receiving care at the primary health care unit serving your neighborhood?*
